# Transcriptomic Insights into the Molecular Mechanisms of Indole Analogues from the *Periplaneta americana* Extract and Their Therapeutic Effects on Ulcerative Colitis

**DOI:** 10.3390/ani15010063

**Published:** 2024-12-30

**Authors:** Yuchen Xie, Qi Yu, Shun Yao, Rui Peng, Jing Li

**Affiliations:** 1 Key Laboratory of Bio-Resources and Eco-Environment (Ministry of Education), College of Life Sciences, Sichuan University, Chengdu 610065, China; 2 School of Chemical Engineering, Sichuan University, Chengdu 610065, China; cusack@scu.edu.cn

**Keywords:** *Periplaneta americana*, indole analogues, NF-κB, transcriptome, ulcerative colitis

## Abstract

Ulcerative colitis (UC) is an inflammatory disease of the intestinal mucosa, and its incidence is steadily increasing worldwide. As a traditional Chinese medicinal insect, *Periplaneta americana* has been broadly utilized in clinical practice to treat wound healing. The tryptophan (Trp), tryptamine (Try), and 1,2,3,4-tetrahydrogen-β-carboline-3-carboxylic acid (Thcc) identified from *P. americana* concentrated ethanol-extract liquid (PACEL) exhibit significant cell proliferation-promoting and anti-inflammatory effects in the treatment of UC, but the mechanism involved remains obscure. Here, a dextran sulfate sodium (DSS)-induced UC mouse model was used to investigate the efficacy of high/low doses of PACEL, Trp, Try, and Thcc. Transcriptome sequencing was employed to detect the gene expression in the mouse intestine. The results showed that high doses of PACEL, Trp, Try, and Thcc could significantly improve weight loss and diarrhea, notably in the PACEL and Trp groups. Transcriptome analysis indicated that statistically changed genes in four treatment groups were specifically enriched in the immune system. Of these, the integrated analysis identified six hub genes (*IL1β*, *CCL4*, *CXCL5*, *CXCR2*, *LCN2*, and *MMP9*) regulated by NF-κB, which were significantly downregulated. This study investigates the molecular mechanisms underlying the UC treatment properties of indole analogues from PACEL, potentially through the inhibition of the NF-κB signaling pathway.

## Simple Summary

*Periplaneta americana* is a traditional medicinal insect, and its extracts are valued for their precise clinical efficacy. *P. americana* concentrated ethanol-extract liquid (PACEL) contains indole analogues, including tryptophan (Trp), tryptamine (Try), and 1,2,3,4-tetrahydrogen-β-carboline-3-carboxylic acid (Thcc), which exhibit notable biological activities. Using a dextran sulfate sodium (DSS)-induced ulcerative colitis (UC) mouse model, we compared the efficacy of PACEL, Trp, Try, and Thcc in mitigating UC symptoms. Transcriptome sequencing was conducted to detect the gene expression in the colon tissues and identify key biological pathways. This study compared the therapeutic effects of PACEL and its three indole analogues in animal experiments and transcriptomic analysis, revealing that Trp, Try, and Thcc in PACEL may exert therapeutic effects by inhibiting NF-κB signaling in the treatment of UC.

## 1. Introduction

Ulcerative colitis (UC) is a chronic, recurrent inflammatory bowel disorder affecting the colon [1]. The increasing incidence of UC in recent years has substantially impacted the quality of life [2]. Consequently, there is an urgent need to develop more effective natural therapeutic agents [3,4,5]. The complicated pathophysiology of UC remains unclear and involves dysfunctional immune responses, as well as environmental and genetic factors, complicating drug development [6,7]. A large body of evidence has shown that inflammatory cytokines play significant roles in UC by regulating the immune response [8,9]. Genes involved in the nuclear factor–kappa B (NF-κB) signaling pathway are largely associated with susceptibility to colon inflammation [10]. Moreover, studies found that some effective components of medical plants or insects exhibit efficacy in treating UC, such as baicalin extracted from the dried root of *Scutellaria baicalensis* Georgi [11,12], astragalus polysaccharides extracted from *Astragalus membranaceus* (Fisch.) Bunge [13], and curcumin derived from a rhizome of turmeric (*Curcuma longa* L.) [14]. Natural product preparations like traditional Chinese medicine have emerged as a therapeutic approach for UC due to their effectiveness and safety profile [15].

*Periplaneta americana*, as an important medicinal insect, has high therapeutic value with antibacterial [16], antioxidant [17], and anti-inflammatory activities [18], and nutritional value as food and feed for humans and livestock [19]. Based on *P. americana*, clinical drugs such as Kangfuxin oral liquid have been developed, which are widely used in the treatment of mucositis [20], pulmonary fibrosis [21], and recurrent oral ulcers [22]. Different from biological agents with toxic and adverse reactions, the extract of *P. americana* can improve intestinal healing and repair mucosal damage without side effects, but its protective mechanisms are complex and not yet fully understood [23]. Meanwhile, the primary active compounds of *P. americana* in treatment were not confirmed due to the difficulty in isolating a single effective component from complex extracts. In our previous study, we used the high-performance liquid chromatography (HPLC) method to determine the five major chromatographic peaks of *P. americana* concentrated ethanol-extract liquid (PACEL) and found, for the first time, high content of tryptophan and abundant β-carbolines that effectively inhibited pro-inflammatory cytokines and exhibited good anti-inflammatory effects [24]. However, the mechanisms underlying UC treatment of these indole analogues warrant further investigation. Therefore, based on PACEL and three identified indole analogues, including L-tryptophan (Trp), tryptamine (Try), and 1,2,3,4-tetrahydro-β-carboline-3-carboxylic acid (Thcc), this study aimed to evaluate the biological activities and molecular mechanisms of PACEL and its indole analogues in promoting mucosal repair using transcriptomic analysis. We used a 4% dextran sulfate sodium (DSS) solution to construct a UC mouse model and verified the precise mechanisms underlying UC treatment by RNA-seq at the gene expression level.

## 2. Materials and Methods

### 2.1. Experimental Animals

The 8-week-old SPF-grade ICR male mice were purchased from Chengdu Dossy Co., Ltd. (Chengdu, China) and fed in the IVC ventilation cages of the SPF animal lab at the College of Life Sciences of Sichuan University. The mice were exposed to 12 h:12 h light/dark cycle light (22–24 °C), with ad libitum access to food and water. Regular cleaning of drinking water containers and cages. Each cage received a consistent daily supply of water and nutritionally standard laboratory feed (Chengdu Dossy Co., Ltd., Chengdu, China), which comprised a fixed formulation of animal protein, plant protein, and carbohydrates.

### 2.2. Reagents and Equipment

The *Periplaneta americana* concentrated extract liquid (PACEL) (containing 2 g worms/mL) was provided by Good Doctor Company (Chengdu, China) for experiments on ulcerative colitis in mice. L-tryptophan (Trp) and tryptamine (Try) were purchased from Shanghai Shenggong Bioengineering Technology Service Co., Ltd. (Shanghai, China), and 1,2,3,4-tetrahydro-β-carboline-3-carboxylic acid (Thcc) was purchased from Shanghai McLean Biochemistry Co., Ltd. (Shanghai, China). DSS (molecular weight 36,000–50,000) was purchased from Yeasen Biotechnology Co., Ltd. (Shanghai, China). Ultrapure water was obtained by a water purification system UPH-1-10T (ULUPURE, Chengdu, China).

The ultrapure water meter was purchased from Thermo Fisher Scientific, Inc. (Waltham, MA, USA). Digital panoramic scanner (non-fluorescent) Wisleap WS-10 was sourced from WISLEAP medical technology (Jiangsu) Co., Ltd., Rugao, China.

### 2.3. UC Mice Molding and Grouping

The acute UC model in mice was induced by sodium dextran sulfate (DSS) [25]. DSS was dissolved in ddH_2_O to prepare a 4% DSS (*w*/*w*) solution every day and provided to experimental mice instead of drinking water for 8 days. The mice in the Control group were given only ddH_2_O. After successful UC model induction, the UC mice were treated with high or low dosages (HD or LD) of three indole analogues (Trp, Try, and Thcc) and PACEL to investigate their reparative effects. A total of 10 experimental groups were established, with four mice randomly assigned to each group. The 10 groups included the Control group (without DSS treatment), Model group, PACEL-HD/LD group, Trp-HD/LD group, Try-HD/LD group, and Thcc-HD/LD group. Concentrations of PACEL, Trp, Try, and Thcc were 2 g worms/mL, 10 mg/mL, 30 mg/mL, and 1 mg/mL, respectively. The concentration settings were established based on our previous studies [24]. From day 9 to day 16, a high dose (300 μL) or low dose (100 μL) of the four drugs was intragastrically administrated to the UC mice, while the Control and Model mice were treated with ddH_2_O.

### 2.4. Disease Activity Index (DAI) and Histologic Analysis

During the experiments, the changes in body weight of the mice were recorded. At the same time, mortality, stool consistency, and hematochezia were observed every day [26]. The disease activity index (DAI) is an important indicator of the severity of the disease. When mice develop colitis, the higher the DAI index, the more severe the body damage situation, indicating serious tissue damage in mice. The parameters for DAI are shown in Table 1. On day 16, all mice were euthanized by cervical dislocation and dissected. The colon tissues were cut and fixed with 4% paraformaldehyde to prepare paraffin sections, with a portion reserved for RNA sequencing. The tissues were dehydrated, embedded in paraffin, sectioned at 5 μm, stained with HE, and observed microscopically [27]. The Wisleap WS-10 image analysis system was used to collect picture information.

### 2.5. DEG Analysis, GSEA, and WGCNA

Three mice from each group were randomly selected to collect colon tissues for high-throughput transcriptome sequencing. After deleting low-quality reads from raw sequence data (Raw reads) and joint adapter sequence, clean sequence data (Clean reads) were obtained by quality filtering of FastQC v0.12.0 (https://www.bioinformatics.babraham.ac.uk/projects/fastqc/, accessed on 2 October 2023) and Trimmomatic v0.36 [28]. Then, Clean reads were aligned to the reference genome Mus_musculus_CRCm39 (http://ftp.ensembl.org/pub/release-107/fasta/mus_musculus/dna/, accessed on 2 October 2023) using the HISAT2 v2.1.0 [29]. Using featureCounts v2.0.1 [30] to obtain expression matrix files. Subsequently, Fragments Per Kilobase of transcript per Million mapped reads (FPKM) were used to normalize the expression level, while DESeq2 [31] was used for screening differentially expressed genes between different groups, and the thresholds were |log2FC| ≥ 1 and *p.adj* (adjusted *p*-value) < 0.05.

The possible role of the diagnostic genes was determined using the gene set enrichment analysis (GSEA) [32]. Weighted gene co-expression network analysis (WGCNA) was used to identify important modules and to acquire hub genes [33]. The scale-free network features were constructed when the power of β = 3 (R^2^ = 0.85). Hub genes were extracted according to the correlation (MM) between gene expression and gene module characteristic genes (eigengene) and the absolute correlation value (GS) between genes and phenotype. The screening threshold was MM > 0.8 and GS > 0.2. Gene clusters in the modules were derived via the exportNetworkToCytoscape function for subsequent visualization.

Gene ontology (GO) and Kyoto encyclopedia of genes and genomes (KEGG) pathway enrichment analysis of the resulting DEGs, GSEA gene sets, and hub genes were analyzed using the R package “clusterProfiler” [34], and the visualization work was performed using the R package “ggplot2” [35]; *p.adj* (adjusted *p*-value) < 0.05 was considered significant. GraphPad (Prism 8.0.2) was applied for data statistics and one-way ANOVA.

### 2.6. PPI Network Analysis

The STRING database [36] was used to generate a protein–protein interaction (PPI) network by downloading interactions with medium confidence scores ≥ 0.4. Then, Cytoscape v3.8.2 [37] was employed in network biology analysis and visualization. To identify significant gene clusters and hub genes, the Molecular Complex Detection (MCODE) algorithm was employed (K core = 2, degree cutoff = 2, max depth = 100, and node score cutoff = 0.2). Additionally, the cytoHubba-MCC plugins, with default parameters, were utilized for the same purpose [38]. Furthermore, the iRegulon plugin (v1.3), employing default cutoff criteria, was utilized to screen for key transcription factors (TFs) [39].

### 2.7. ceRNA Network Construction

In the ceRNA network, we used microRNAs (miRNAs) to link target hub genes to their respective long non-coding RNAs (lncRNAs). miRDB, DIANA-microT v5.0, and miRWalk 3.0 with default parameters were used to predict the miRNAs [40,41,42]. StarBase (v3.0) was utilized with high stringency to predict miRNA-lncRNA interactions [43]. Cytoscape v3.9.1 was employed to construct and visualize interaction networks [37].

## 3. Results

### 3.1. PACEL, Trp, Try, and Thcc Treatment Ameliorated DSS-Induced Mice Colitis

**Figure 1:**
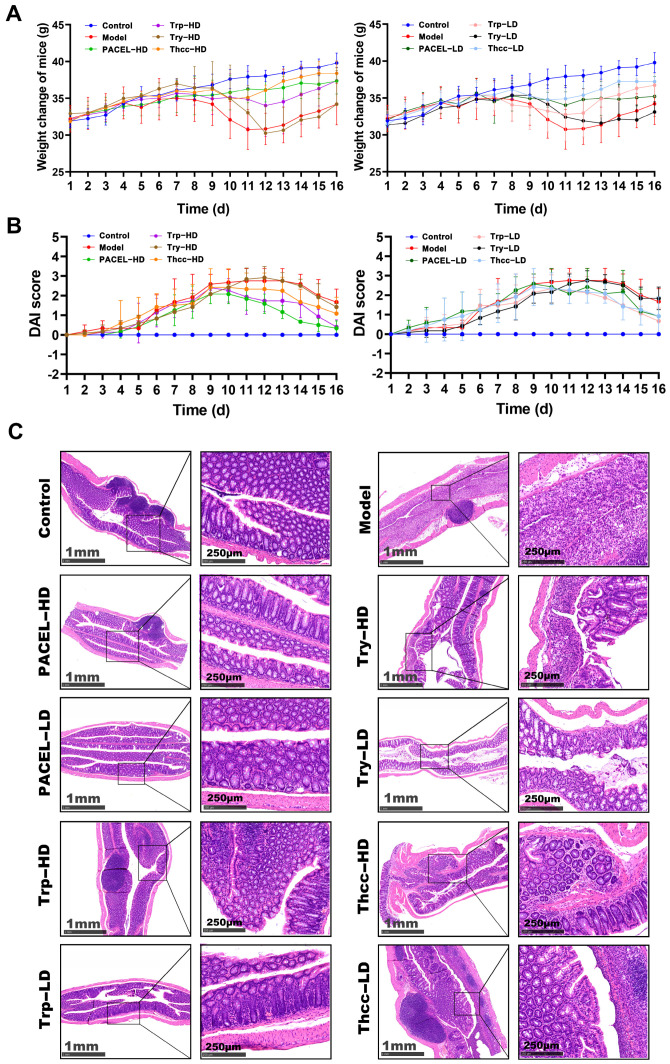
PACEL, Trp, Try, and Thcc treatment alleviated the symptoms of DSS-induced UC in mice: (**A**) mice’s body weight change during experiment (n = 4); (**B**) disease activity index (DAI) scores; and (**C**) colon histological section with hematoxylin–eosin staining.

UC model mice were induced by DSS. The treated mice developed mental depression, reduced body mass, diarrhea, and hematochezia compared to the Control group given ddH_2_O throughout. The DAI scores continuously increased to 3 points in 8 days, indicating successful modeling of UC [25,44]. After the oral administration of PACEL and three indole analogues, both the HD and LD groups of mice showed less weight loss than the Model group and began to regain weight from day 12 (Figure 1A). Concurrently, the alleviation of symptoms, including depression, diarrhea, and blood stool, resulted in a reduction in the DAI scores (Figure 1B). H&E histopathological analysis further demonstrated that PACEL, Trp, Try, and Thcc could alleviate colon tissue damage in the UC model mice (Figure 1C). In the Control group without DSS treatment, no inflammatory cells were observed, and the tissue structure was intact. In the Model group, colitis mice developed typical crypt damage, which is embodied in the massive infiltration of inflammatory cells in the intestinal mucosal layer. Compared with the Model group, both the HD and LD of drug-treated groups had mild infiltration of inflammatory cells, showing significant improvements in colon tissue damage and pathological presentations. In particular, the improvement was more notable in the PACEL-HD and Trp-HD groups than in the other groups.

### 3.2. Identification of DEGs and Functional Enrichment Analysis

**Figure 2:**
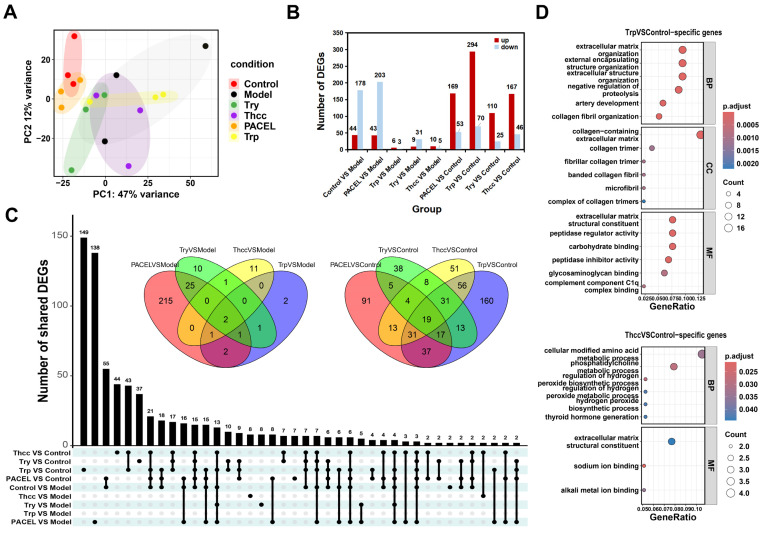
Analysis of gene expression profiling results: (**A**) principal component analysis (PCA); (**B**) quantitative results of DEGs analysis; (**C**) Tte Venn plots and UpSetR plot of 9 groups DEGs; and (**D**) GO enrichment of the unique DEGs in the Trp and Thcc groups compared to the Control group.

RNA-seq analysis of colonic tissue was performed to investigate the transcriptomic changes between the Control group, Model group, and the high doses of drug-treated groups (n = 3). The sequencing generated the averaged raw bases of 7.51 Gb and averaged clean bases of 7.02 Gb in each sample, with Q30% in the range of 91.15% to 93.30% (Table 2). After standardizing the gene expression results calculated by featureCounts [30], the PCA analysis showed that the PACEL group was closest to the Control group in gene expression level. Moreover, the distinction between the Try and Control groups was less conspicuous at the PC1 level, while the Trp group demonstrated a closer alignment with the Control group at the PC2 level (Figure 2A). The gene expression heatmap of DEGs showed that there were significant differences between all treatment groups and the Model group (Figure S1). Compared with the Model group, 222, 246, 9, 40, and 15 DEGs were significantly regulated in the Control, PACEL, Trp, Try, and Thcc groups, respectively. Furthermore, the four drug treatment groups shared two differentially expressed genes, *CYP2e1* and *Vit*. When compared to the Control group, 222, 364, 135, and 213 DEGs were identified in the groups PACEL, Trp, Try, and Thcc, respectively, with 19 genes overlapping across four groups (Figure 2B,C). The KEGG and GO enrichment results of DEGs indicated that all four treatment groups exhibited significant commonalities in enrichment profiles, including the KEGG pathways “cytokine–cytokine receptor interaction”, “IL-17 signaling pathway”, “TNF signaling pathway”, and “inflammatory bowel disease (IBD)”, and the GO pathways “immune receptor activity”, “IgG immunoglobulin complex”, and “CXCR chemokine receptor binding”, which were regarded as crucial effects on inflammation. Additionally, the lipid metabolism-related pathways were noted to appear in the KEGG and GO pathway enrichment results of comparison of the four treatment groups and Model group (Figures S2 and S3). Enrichment analysis of the 160 specific DEGs in the Trp vs. Control revealed significant associations with pathways in the regulation of collagen-related processes and extracellular matrix organization, including “collagen-containing extracellular matrix” and “collagen fibril organization”. In addition, the Thcc group has unique enriched entries in the modification and transformation of amino acids (Figure 2D).

Further analysis found that 83, 3, 24, and 4 shared genes can be obtained from the intersection of DEGs in the group PACEL, Trp, Try, and Thcc. KEGG enrichment results of shared DEGs showed that PACEL treatment mainly affected inflammatory pathways, Trp and Thcc treatment mainly affected drug metabolism, and Try treatment mainly affected protein and fat digestion. GO molecular function analysis demonstrated that digestive tract and immune system relative pathways, such as serine-type peptidase activity, were correlated with therapeutic functions of PACEL, Trp, and Try (Figure S4).

### 3.3. Treatment Altered Transcriptional Profiles of Colons in UC Model Mice

**Figure 3:**
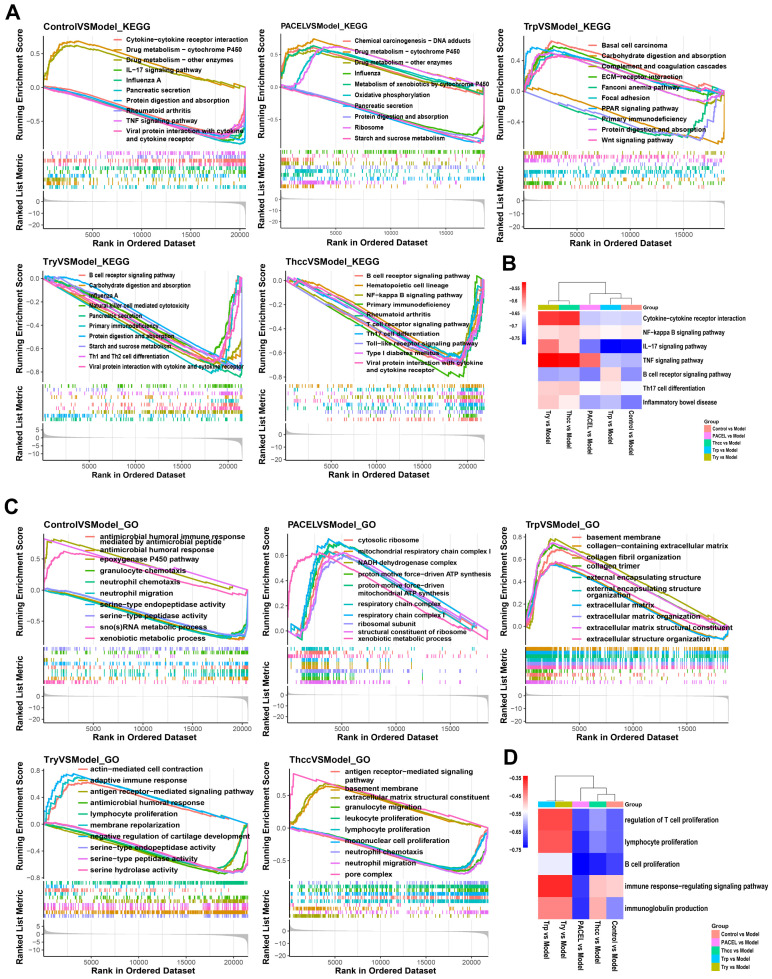
Gene set enrichment analysis (GSEA) analysis: (**A**) KEGG analysis of five groups; (**B**) the heatmap of KEGG enrichment pathways; (**C**) GO analysis of five groups; and (**D**) the heatmap of GO enrichment terms.

GSEA analysis indicated that the top annotated collection of genes was enriched in the immune system, suggesting that immune responses may play a critical role in UC recovery. In the PACEL group, oxidative phosphorylation and drug metabolism cytochrome P450 pathway were the KEGG pathways enriched by GSEA gene sets, and the GO keywords included NADH dehydrogenase complex and ATP synthesis. In the treatment of Trp, the GO keywords included collagen fibril organization and extracellular matrix structural constituent, and the KEGG analysis showed that these genes were highly associated with the wnt signaling pathway, primary immunodeficiency, and the PPAR signaling pathway. The B cell receptor signaling pathway, Th1 and Th2 cell differentiation, and natural killer cell-mediated cytotoxicity were shown to be significant KEGG terms for Try GSEA gene sets. The GO pathways of Try GSEA gene sets were shown to be abundant in adaptive immune response, antimicrobial humoral response, and lymphocyte proliferation. In the treatment of Thcc, the nuclear factor–kappa B (NF-κB) signaling pathway, T cell receptor signaling pathway, and B cell receptor signaling pathway were shown to be significant KEGG terms, and the GO pathways were abundant in the antigen receptor-mediated signaling pathway, leukocyte proliferation, and neutrophil chemotaxis (Figure 3A,C). Functional enrichment analysis revealed that the GSEA gene sets of PACEL and the three indole compounds were associated with immunology, endoplasmic reticulum (ER), and metabolism. Based on running enrichment scores, the heatmaps of KEGG (Figure 3B) and GO (Figure 3D) co-enrichment pathways revealed distinct patterns of enrichment. Notably, the enrichment scores for the NF-κB signaling pathway exhibited relative uniformity across all groups. The enrichment scores for the IL-17 signaling pathway were more closely aligned between the PACEL and Trp groups, as well as between the Thcc and Try groups.

### 3.4. Co-Expression Modules and Hub Genes Analysis

**Figure 4:**
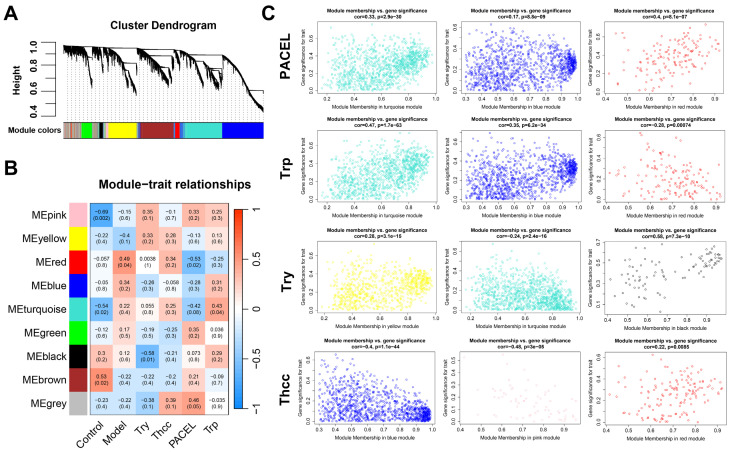
Weighted gene co-expression network analysis (WGCNA) analysis: (**A**) The hierarchical clustering tree shows the network and the nine identified modules. (**B**) Module–trait relationships in different groups. Heatmap plot shows the adjacencies in the eigengene network. The relevant *p*-value and correlation coefficient are listed in each cell. (**C**) The scatterplot of gene significance (GS) versus module membership (MM) in the top three modules in four groups.

WGCNA was employed to construct and analyze networks with active associations, using Spearman correlation to identify significant modules. The WGCNA analysis identified a total of nine modules in which genes had similar co-expression characteristics (Figure 4A,B). Nine modules were labeled in nine different colors. The turquoise, blue, and red modules were selected as crucially important modules both in the PACEL and Trp groups. Genes in the yellow, turquoise, and black modules were highly correlated with Try, while important modules associated with Thcc were blue, pink, and red modules (Figure 4C). In addition, 54, 3, 18, and 4 overlapping DEGs were respectively identified as the PACEL, Trp, Try, and Thcc hub genes (Figure S5, Table S1).

### 3.5. Inhibition of NF-κB Activity Suppressed the Expression of Hub Genes and Alleviated UC

**Figure 5:**
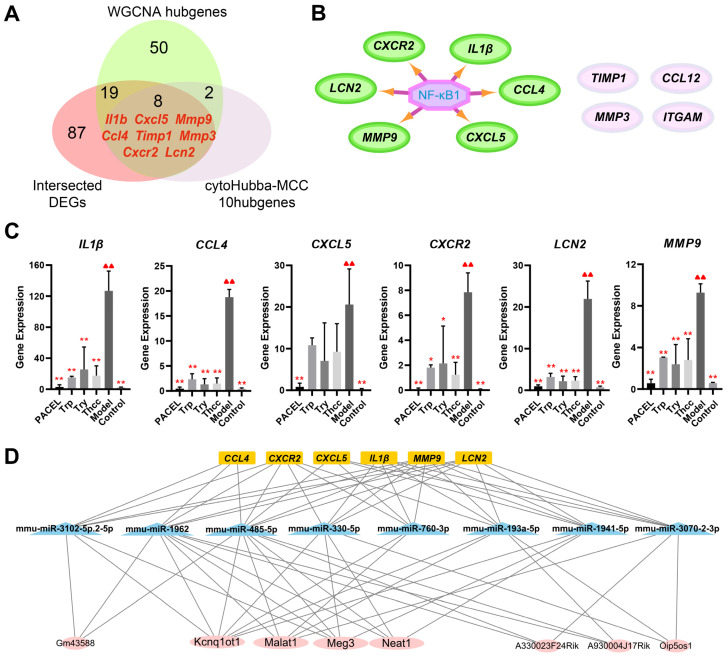
(**A**) Venn diagram of the overlapping genes. (**B**) The master regulator predicted by the iRegulon tool is highlighted in purple, and target genes are in green, while unregulated genes are in pink. (**C**) Differential gene expression analysis in DEGs analysis (** *p* < 0.001, * *p* < 0.05, compared with the Model group. ▲▲ *p* < 0.001, compared with the Control group). (**D**) The center of the lncRNA-miRNA-hub genes network, including eight lncRNAs. Yellow: hub gene. Blue: miRNA. Pink: lncRNA.

An interaction network comprising 28 nodes and 111 edges was constructed from the proteins encoded by 79 hub genes, which were derived from the combination of four treatment groups (Figure S6A). After filtration by the MCODE plugin, all modules were divided into two clusters (cluster 1, score = 7.8, 11 nodes, and 39 edges; cluster 2, score = 3.667, 7 nodes, and 11 edges) (Figure S6B). We identified 10 cytoHubba-MCC-hub genes (score > 900) (Figure S6C), which intersected eight genes with WGCNA hub genes and shared DEGs, including *IL1β*, *CCL4*, *CXCL5*, *CXCR2*, *LCN2*, *MMP9*, *MMP3*, and *TIMP1* (Figure 5A). Using the iRegulon plugin, TF binding motifs were tested for eight hub genes associated with UC traits, revealing that six genes, *IL1β*, *CCL4*, *CXCL5*, *CXCR2*, *LCN2*, and *MMP9*, were regulated by NF-κB1 (Figure 5B). Subsequently, the differential expression analysis of the six specifically expressed hub genes revealed significant downregulation of mRNA expression levels in the PACEL, Trp, Try, and Thcc groups when compared to the Model samples (Figure 5C).

### 3.6. CeRNA Network Construction

Gene expression is regulated by epigenetic factors, such as miRNAs and lncRNAs [45]. To identify miRNAs that regulate the activities of six hub genes, we obtained 90 regulatory miRNAs from three miRNA databases, where miRNA mmu-miR-485-5p exhibited the highest number of associated genes (Figure S7A). In addition, we investigated the lncRNAs that interacted with regulator miRNAs and obtained eight sub-networks (Figure S7B). Notably, we found that the lncRNA KCNQ1OT1 mediated crosstalk between seven miRNAs, MALAT1 and MEG3 mediated crosstalk between five miRNAs, and NEAT1 mediated crosstalk between four miRNAs (Figure 5D). Taken together, the ceRNA network may influence the expression of six hub genes alongside NF-κB1.

## 4. Discussion

As a traditional Chinese medicinal material, the extract of *P. americana* possesses significant pharmacological value, with notable effectiveness in wound healing and repair [46]. Previously, we identified tryptophan (Trp) and three indole analogues in PACEL for the first time, including tryptamine (Try) and 1,2,3,4-tetrahydro-β-carboline-3-carboxylic acid (Thcc), and found these indole analogues showed good proliferative and anti-inflammatory activities, which likely contribute to wound healing in UC mice [24]. However, the molecular mechanism of these indole analogues on wound healing in UC mice is yet to be uncovered, which is critical in their potential for therapeutic applications. Further investigations are essential to delineate the specific pathways and targets involved in the biological activities of these indole derivatives.

In this study, we compared the therapeutic effects of different dosages of PACEL and three indole analogues on DSS-induced UC mice. UC causes severe inflammation of the colorectal mucosa, and its treatment involves complex processes, such as mucosal healing, inflammatory response, cell proliferation, and migration [47]. After oral ingestion of PACEL or the indole analogues, increased body weight and decreased DAI score were observed, indicating that these compounds could improve the symptoms in UC mice. High dosages of four treatments exhibited better effects than low dosages, with PACEL and Trp showing the best repairing effect. Meanwhile, pathological results showed that a high dosage of PACEL and the indole analogues improved the disappeared mucosal crypt, destroyed submucosa, and neutrophil aggregation, suggesting that they could promote colonic mucosal repair in UC mice. Indole is widely considered one of the most promising heterocyclic moieties, and its derivatives have been extensively utilized in the field of drug development [48]. Among them, β-caroline alkaloids are a large group of natural and synthetic indole alkaloids with a variety of pharmacological activities, including tumor suppression, antiviral, and antioxidant effects [49,50]. In addition, tetrahydro-β-carboline is the key structural unit with important physiological activities and an essential structure for some antitumor drugs and active compounds [51,52]. For example, Thcc has been reported to induce apoptosis in HCT-8 cancer cells [53]. Previously, Thcc was usually reported to be present in plants [54] and food [55] and showed antioxidant capacity [56], whereas in PACEL, it may be a Trp analogue produced through a similar synthetic pathway [57]. Identification of key enzymes involved in the conversion of Trp and indole analogues is important for further understanding either biosynthetic mechanisms or metabolic pathways.

Transcriptome analysis demonstrated that compared with the Model group, the GO enrichment analysis of DEGs and GSEA gene sets in the PACEL and three indole analogues treatment groups were functionally enriched in the items related to the immune system, metabolic processes, and defense response, indicating that mucosal immunity may play an important role in colon inflammatory injury and treatment process. Furthermore, the results of KEGG enrichment revealed that the four treatments for UC were not only related to cytokine–cytokine receptor interaction, TNF signaling, IL-17 signaling, the drug metabolism cytochrome P450, and B/T cell receptor signaling pathways but also involved lipid metabolism. Notably, all the treatment groups shared the DEGs *CYP2e1* and *Vit. CYP2e1* belongs to the cytochrome P450 (CYPs) family, which mediates the metabolism of most drugs [58]. Kusunoki et al. found that the gene expression of *CYP3a11*, *CYP1a2*, *CYP2c29*, *CYP2d9*, and *CYP2e1* in the liver declined with the deterioration of UC, reaching a minimum on day 10 [59]. The main reason for the reduced expression level of *CYPs* may be related to the increased mucosal permeability and inflammatory cytokines caused by colonic inflammation [60]. These results suggest that the PACEL and three indole compounds could improve intestinal mucosal damage and inflammation, and more attention should be paid to the role of *CYP* genes. The *Vit* gene encodes vitrin, an extracellular matrix (ECM) protein that plays a vital role in cell adhesion and migration [61,62]. These functions are essential for matrix assembly, where tissue remodeling and cellular interactions are critical for healing and inflammation resolution in UC treatment. While the three indole compounds exhibit similarities in immune regulation, their transcriptomic results also demonstrate notable differences. A study with over 500 IBD patients found a negative correlation between serum Trp levels and disease activity, suggesting that Trp deficiency and altered metabolism may contribute to IBD pathogenesis [63]. Moreover, dietary Trp supplementation in a mouse model enhanced serum amino acid profiles, intestinal immunity, and gut microbiota [64]. Specifically, the number of DEGs in the Trp group was higher than that in the Try and Thcc groups, indicating that the Trp group may possess greater specificity and influence in biological regulation. Additionally, the unique DEGs identified in the Trp group are closely associated with collagen production and extracellular matrix remodeling, suggesting that this group may effectively promote tissue repair and remodeling by enhancing collagen synthesis and organization [65]. Trp may play a crucial role in the regulation of extracellular matrix organization and collagen-related functions, which are vital for tissue repair, regeneration, and immune modulation. As prior research has reported various components within *P. americana* extracts [66], the interactions among these constituents and their impacts on multiple biological activities are not yet fully understood. Thus, further integrative studies on the synergistic effects of molecules within PACEL will be crucial for its clinical application.

Many studies have reported that NF-κB had a crucial effect on the development of UC, and the infiltration of colon immune cells regulated by NF-κB is considered to be one of the causes of UC [67]. Multiple therapeutic drugs have been found to treat UC by affecting the NF-κB pathway [68], such as curcumin [67], probiotics [69], and mesalazine [70]. The activated NF-κB pathway in UC leads to an upregulation in the expression of pro-inflammatory cytokines and adhesion factors while simultaneously inhibiting the production of apoptotic factors [71]. In this study, comprehensive bioinformatics analysis using WGCNA and Cytoscape revealed *IL1β*, *CCL4*, *CXCL5*, *CXCR2*, *LCN2*, and *MMP9* as the critical genes after PACEL and the three indole compounds were treated, and determined the central role of the TF NF-κB regulation. The gene expression results indicated that six genes regulated by NF-κB were all significantly downregulated in the areas of the colon after indole compounds treatment. Among the 6 NF-κB related genes, *IL-1β* was reported to have important effects on regulating innate immune responses, and the levels of IL-1β were significantly increased in colitis [72] as members of the chemotactic cytokines families, *CCL4*, *CXCL5*, and *CXCR2*, play key roles in regulating mucosal inflammation and the immune system by promoting the migration of neutrophils to inflammatory sites and can be used as characteristic genes indicating the degree of inflammation in UC [73,74]. Additionally, *MMP9* [75] expression in inflamed mucosa and serum LCN2 protein [76] can also be reliable biomarkers of disease activity in UC. Significant upregulation of *MMP9* expression in UC patients affects the coagulation factors and the intestinal mucosal permeability, which will further worsen the UC symptoms [77]. Alternatively, the restoration of *CYP2e1* gene expression to normal levels after the treatment may also be due to the inhibition of the NF-κB pathway [78]. Ni et al. detected cytokines expression in mice treated with *P. americana* extract Ento-A by ELISA and flow cytometry, inferring that Ento-A improved UC symptoms by modulating immune response and inhibiting PI3K/AKT/NF-κB pathway [23]. The results of the transcriptome analysis in this study demonstrated that PACEL and its three indole compounds showed inhibitory effects on the NF-κB signaling pathway at the gene expression level. In addition to the pivotal role of NF-κB downregulation, the molecular mechanisms underlying the therapeutic effects in UC may also involve other significant pathways, such as JAK-STAT, MAPK, and TGF-β signaling. Exploring the interactions among these pathways and NF-κB could deepen insights into UC treatment and guide the development of more targeted therapies. The ceRNA network has garnered considerable attention in recent years due to its pivotal role in the regulation of gene expression [79]. We predicted a ceRNA network of the lncRNA-miRNA axis in the regulation of 6 NF-κB related genes. With the most regulated connections in the network, the effect of KCNQ1OT1 on lncRNAs in the development of colon cancer has been confirmed in several studies [80,81]. LncRNA MALAT1 was shown to induce UC by upregulating lncRNA ANRIL [82], and overexpression of MALAT1 inhibited cell viability while targeting miR-30c-5p via the NF-κB/TGF-β/Wnt-β-catenin signaling pathway increased apoptosis and inflammation [83]. Upregulation of lncRNA MEG3 alleviated oxidative stress, inflammatory response, and apoptosis in UC rats [84]. LncRNA NEAT1 was also reported to participate in UC progression by inhibiting miR-493-5p expression [85]. In summary, the predicted lncRNA and key miRNAs, which potentially acted as an upstream regulator, may play important roles in the progression of PACEL to cure UC by targeting the NF-κB pathway, providing new avenues for combination targeted therapy in active UC.

## 5. Conclusions

Building upon the previous identification of indole compounds within PACEL, this study compared the reparative activities of PACEL and three indole compounds (Trp, Try, and Thcc) at different dosages on UC mice, additionally elucidating their molecular mechanisms of action through transcriptome sequencing of colon tissues. In conclusion, PACEL, Trp, Try, and Thcc effectively suppressed the body mass reduction, increased the DAI score and histological damage caused by DSS, and established the overall immune regulation to achieve the immune balance so as to alleviate the inflammatory damage caused by pro-inflammatory factors or anti-inflammatory factors imbalance. PACEL and its active components Trp, Try, Thcc downregulate the gene expression of the NF-κB signaling pathway in UC mice to alleviate the mucosal inflammatory damage caused by inflammatory factor imbalance, protect the integrity of intestinal mucosal barrier and intestinal epithelial cells, and play a core role in repairing intestinal damage, which provides a theoretical basis for the clinical application of PACEL and indole derivatives.

## Figures and Tables

**Table 1:** Scoring system for DAI.

Score	Weight Loss (%)	Fecal Consistency	Hematochezia
0	2	normal	normal
1	1–5	mildly	brown
2	5–10	very soft	
3	10–15	watery	bloody stool
4	>15		

**Table 2:** Sequencing data statistics.

Group	Raw Reads	Raw Bases/Gb	Clean Reads	Clean Bases/Gb	Q20%	Q30%	GC%
Control	42,696,764	6.40	42,310,822	6.07	97.60	92.98	48.83
	56,141,084	8.42	55,660,204	7.89	97.59	92.98	48.25
	44,270,844	6.64	43,841,050	6.26	97.30	92.32	47.89
Model	57,692,332	8.65	57,107,032	8.01	97.44	92.62	48.09
	53,302,866	7.99	52,812,240	7.47	97.60	92.99	48.08
	52,981,578	7.95	52,281,226	7.45	96.77	91.16	47.90
PACEL	56,887,736	8.53	56,328,228	8.06	97.49	92.77	47.78
	44,062,114	6.60	43,558,032	6.14	92.47	92.70	48.27
	52,104,786	7.82	51,465,340	7.29	97.40	92.53	48.42
Trp	45,386,754	6.81	44,906,136	6.41	97.36	92.47	48.30
	53,629,778	8.04	53,098,600	7.52	92.47	92.71	47.86
	51,868,678	7.78	51,333,280	7.24	97.65	93.12	48.13
Try	45,720,034	6.86	45,132,078	6.38	96.92	91.51	48.08
	42,604,908	6.39	42,225,936	6.01	97.72	93.30	48.31
	56,801,716	8.52	56,290,092	7.90	97.54	92.87	48.11
Thcc	45,957,584	6.89	45,504,176	6.43	97.39	92.49	48.30
	53,156,784	7.97	52,652,812	7.45	97.35	92.36	47.92
	45,837,944	6.88	45,392,864	6.38	97.34	92.43	49.18

## Data Availability

The raw data supporting the conclusions of this article will be made available by the authors on request.

## Supplementary Materials

The following supporting information can be downloaded at https://www.mdpi.com/article/10.3390/ani15010063/s1: Figure S1: The heatmap of comparing DEGs expression profile; Figure S2: KEGG pathway enrichment of DEGs in different groups; Figure S3: GO pathway enrichment of DEGs in different groups; Figure S4: The KEGG (up) and GO (down) pathway enrichment of the shared DEGs in different treatment; Figure S5: Venn diagram shows genes identified from the intersection of DEGs and significant module genes in WGCNA; Figure S6: Protein interaction network. (A) PPI network. The nodes represent proteins. The edges represent their interaction. (B) MCODE sub-network, including cluster 1 and cluster 2. (C) Cytohubba-MCC was used to identify hub genes in the network; Figure S7: The integrated lncRNA-miRNA-hub genes network. (A) The miRNA-hub genes network included six hub genes and 90 regulatory miRNAs. (B) The lncRNA-miRNA-hub genes network was constructed, including eight lncRNA-miRNA sub-networks and the crossover points; Table S1: Overlapping genes between DEGs and WGCNA module genes.

## References

[1] Hu Y., Ye Z., Wu M., She Y., Li L., Xu Y., Qin K., Hu Z., Yang M., Lu F. (2021). The Communication between Intestinal Microbiota and Ulcerative Colitis: An Exploration of Pathogene-Sis, Animal Models, and Potential Therapeutic Strategies. Front. Med..

[2] Zheng Y.Y., Wang X., Si J.T., Sun Y.X., Hou W.B., Liu J.P., Li Y.X. (2021). Randomized Clinical Trials of Traditional Chinese Medicines for Treating Ulcerative Colitis: A Scoping Review. World J. Tradit. Chin. Med..

[3] Liu Y., Li B.G., Su Y.H., Zhao R.X., Song P., Li H., Cui X.H., Gao H.M., Zhai R.X., Fu X.J. (2022). Potential Activity of Traditional Chinese Medicine against Ulcerative Colitis: A Review. J. Ethnopharmacol..

[4] Yokoyama Y., Matsuoka K., Kobayashi T., Sawada K., Fujiyoshi T., Ando T., Ohnishi Y., Ishida T., Oka M., Yamada M. (2014). A Large-Scale, Prospective, Observational Study of Leukocytapheresis for Ulcerative Colitis: Treatment Outcomes of 847 Patients in Clinical Practice. J. Crohns. Colitis.

[5] Shen R. (2018). Experimental Research Progress on Traditional Chinese Medicine Treatments for Ulcerative Colitis. Chin. Tradit. Herb. Drugs.

[6] Kobayashi T., Siegmund B., Le Berre C., Wei S.C., Ferrante M., Shen B., Bernstein C.N., Danese S., Peyrin-Biroulet L., Hibi T. (2020). Ulcerative Colitis. Nat. Rev. Dis. Primers.

[7] Ungaro R., Mehandru S., Allen P.B., Peyrin-Biroulet L., Colombel J.F. (2017). Ulcerative Colitis. Lancet.

[8] Hibi T., Ogata H. (2006). Novel Pathophysiological Concepts of Inflammatory Bowel Disease. J. Gastroenterol..

[9] Tatiya-aphiradee N., Chatuphonprasert W., Jarukamjorn K. (2018). Immune Response and Inflammatory Pathway of Ulcerative Colitis. J. Basic Clin. Physiol. Pharmacol..

[10] Xu M., Kong Y., Chen N., Peng W., Zi R., Jiang M., Zhu J., Wang Y., Yue J., Lv J. (2022). Identification of Immune-Related Gene Signature and Prediction of CeRNA Network in Active Ulcerative Colitis. Front. Immunol..

[11] Ganguly R., Gupta A., Pandey A.K. (2022). Role of Baicalin as a Potential Therapeutic Agent in Hepatobiliary and Gastrointestinal Disorders: A Review. World J. Gastroenterol..

[12] Wang X., Xie L., Long J., Liu K., Lu J., Liang Y., Cao Y., Dai X., Li X. (2022). Therapeutic Effect of Baicalin on Inflammatory Bowel Disease: A Review. J. Ethnopharmacol..

[13] Zhong Y., Xiao Q., Kang Z., Huang J., Ge W., Wan Q., Wang H., Zhou W., Zhao H., Liu D. (2022). Astragalus Polysaccharide Alleviates Ulcerative Colitis by Regulating the Balance of Tfh/Treg Cells. Int. Immunopharmacol..

[14] Sardou H.S., Vosough P.R., Abbaspour M., Akhgari A., Sathyapalan T., Sahebkar A. (2023). A Review on Curcumin Colon-Targeted Oral Drug Delivery Systems for the Treatment of Inflammatory Bowel Disease. Inflammopharmacology.

[15] Zhang X., Zhang L., Chan J.C.P., Wang X., Zhao C., Xu Y., Xiong W., Chung W.C., Liang F., Wang X. (2022). Chinese Herbal Medicines in the Treatment of Ulcerative Colitis: A Review. Chin. Med..

[16] Ali S.M., Siddiqui R., Ong S.-K., Shah M.R., Anwar A., Heard P.J., Khan N.A. (2016). Identification and Characterization of Antibacterial Compound(S) of Cockroaches (*Periplaneta americana*). Appl. Microbiol. Biotechnol..

[17] Nguyen T.T., Chen X., Chai J., Li R., Han X., Chen X., Liu S., Chen M., Xu X. (2020). Antipyretic, Anti-Inflammatory and Analgesic Activities of *Periplaneta americana* Extract and Underlying Mechanisms. Biomed. Pharmacother..

[18] Li X., Liu Y., Song H., Zhao M., Song Q. (2023). Antioxidant, Antibacterial, and Anti-Inflammatory *Periplaneta americana* Remnant Chitosan/Polysaccharide Composite Film: In Vivo Wound Healing Application Evaluation. Int. J. Biol. Macromol..

[19] Boate U.R., Suotonye B.D. (2020). Cockroach (*Periplaneta americana*): Nutritional Value as Food and Feed for Man and Livestock. Asian Food Sci. J..

[20] Luo Y., Feng M., Fan Z., Zhu X., Jin F., Li R., Wu J., Yang X., Jiang Q., Bai H. (2016). Effect of *Kangfuxin* Solution on Chemo/Radiotherapy-Induced Mucositis in Nasopharyngeal Carcinoma Patients: A Multicenter, Prospective Randomized Phase III Clinical Study. Evid. Based Complement. Altern. Med..

[21] Yao H., Wei S., Xiang Y., Wu Z., Liu W., Wang B., Li X., Xu H., Zhao J., Gao Y. (2019). Kangfuxin Oral Liquid Attenuates Bleomycin-Induced Pulmonary Fibrosis via the TGF-*β*1/Smad Pathway. Evid. Based Complement. Altern. Med..

[22] Ma P.T., Wu N.N., Pei R. (2018). Effect of Kangfuxin Liquid Combined with Garlicin Capsules in Treatment of Children with Recurrent Oral Ulcer and on Immune Regulation. J. Stomatol..

[23] Ni L., Lu Q., Tang M., Tao L., Zhao H., Zhang C., Yu Y., Wu X., Liu H., Cui R. (2022). *Periplaneta americana* Extract Ameliorates Dextran Sulfate Sodium-Induced Ulcerative Colitis via Immunoregulatory and PI3K/AKT/NF-ΚB Signaling Pathways. Inflammopharmacology.

[24] Xie Y., Liang S., Zhang Y., Wu T., Shen Y., Yao S., Li J. (2023). Discovery of Indole Analogues from *Periplaneta americana* Extract and Their Activities on Cell Proliferation and Recovery of Ulcerative Colitis in Mice. Front. Pharmacol..

[25] Jialing L., Yangyang G., Jing Z., Xiaoyi T., Ping W., Liwei S., Simin C. (2020). Changes in Serum Inflammatory Cytokine Levels and Intestinal Flora in a Self-Healing Dextran Sodium Sulfate-Induced Ulcerative Colitis Murine Model. Life Sci..

[26] Zhang L., Yao X., Ma M., Ding Y., Zhang H., He X., Song Z. (2021). Protective Effect of L-Theanine against DSS-Induced Colitis by Regulating the Lipid Metabolism and Reducing Inflammation via the NF-ΚB Signaling Pathway. J. Agric. Food Chem..

[27] Li C., Ai G., Wang Y., Lu Q., Luo C., Tan L., Lin G., Liu Y., Li Y., Zeng H. (2020). Oxyberberine, a Novel Gut Microbiota-Mediated Metabolite of Berberine, Possesses Superior Anti-Colitis Effect: Impact on Intestinal Epithelial Barrier, Gut Microbiota Profile and TLR4-MyD88-NF-ΚB Pathway. Pharmacol. Res..

[28] Bolger A.M., Lohse M., Usadel B. (2014). Trimmomatic: A Flexible Trimmer for Illumina Sequence Data. Bioinformatics.

[29] Kim D., Langmead B., Salzberg S.L. (2015). HISAT: A Fast Spliced Aligner with Low Memory Requirements. Nat. Methods.

[30] Liao Y., Smyth G.K., Shi W. (2013). FeatureCounts: An Efficient General Purpose Program for Assigning Sequence Reads to Genomic Features. Bioinformatics.

[31] Love M.I., Huber W., Anders S. (2014). Moderated Estimation of Fold Change and Dispersion for RNA-Seq Data with DESeq2. Genome Biol..

[32] Subramanian A., Tamayo P., Mootha V.K., Mukherjee S., Ebert B.L., Gillette M.A., Paulovich A., Pomeroy S.L., Golub T.R., Lander E.S. (2005). Gene Set Enrichment Analysis: A Knowledge-Based Approach for Interpreting Genome-Wide Expression Profiles. Proc. Natl. Acad. Sci. USA.

[33] Langfelder P., Horvath S. (2008). WGCNA: An R Package for Weighted Correlation Network Analysis. BMC Bioinform..

[34] Wu T., Hu E., Xu S., Chen M., Guo P., Dai Z., Feng T., Zhou L., Tang W., Zhan L. (2021). ClusterProfiler 4.0: A Universal Enrichment Tool for Interpreting Omics Data. Innovation.

[35] Wickham H. (2011). Ggplot2. Wiley Interdiscip. Rev. Comput. Stat..

[36] Szklarczyk D., Gable A.L., Nastou K.C., Lyon D., Kirsch R., Pyysalo S., Doncheva N.T., Legeay M., Fang T., Bork P. (2020). The STRING Database in 2021: Customizable Protein–Protein Networks, and Functional Characterization of User-Uploaded Gene/Measurement Sets. Nucleic Acids Res..

[37] Smoot M.E., Ono K., Ruscheinski J., Wang P.L., Ideker T. (2010). Cytoscape 2.8: New Features for Data Integration and Network Visualization. Bioinformatics.

[38] Chin C.H., Chen S.H., Wu H.H., Ho C.W., Ko M.T., Lin C.Y. (2014). CytoHubba: Identifying Hub Objects and Sub-Networks from Complex Interactome. BMC Syst. Biol..

[39] Janky R., Verfaillie A., Imrichová H., Van de Sande B., Standaert L., Christiaens V., Hulselmans G., Herten K., Naval Sanchez M., Potier D. (2014). IRegulon: From a Gene List to a Gene Regulatory Network Using Large Motif and Track Collections. PLoS Comput. Biol..

[40] Wong N., Wang X. (2014). MiRDB: An Online Resource for MicroRNA Target Prediction and Functional Annotations. Nucleic Acids Res..

[41] Paraskevopoulou M.D., Georgakilas G., Kostoulas N., Vlachos I.S., Vergoulis T., Reczko M., Filippidis C., Dalamagas T., Hatzigeorgiou A.G. (2013). DIANA-MicroT Web Server V5.0: Service Integration into MiRNA Functional Analysis Workflows. Nucleic Acids Res..

[42] Dweep H., Sticht C., Pandey P., Gretz N. (2011). MiRWalk–Database: Prediction of Possible MiRNA Binding Sites by “Walking” the Genes of Three Genomes. J. Biomed. Inform..

[43] Li J.H., Liu S., Zhou H., Qu L.H., Yang J.H. (2014). StarBase V2.0: Decoding MiRNA-CeRNA, MiRNA-NcRNA and Protein–RNA Interaction Networks from Large-Scale CLIP-Seq Data. Nucleic Acids Res..

[44] Travis S.P.L., Higgins P.D.R., Orchard T., Van Der Woude C.J., Panaccione R., Bitton A., O’Morain C., Panés J., Sturm A., Reinisch W. (2011). Review Article: Defining Remission in Ulcerative Colitis. Aliment. Pharmacol. Ther..

[45] Sayed D., Abdellatif M. (2011). MicroRNAs in Development and Disease. Physiol. Rev..

[46] Liang S., Zhang Y., Li J., Yao S. (2022). Phytochemical Profiling, Isolation, and Pharmacological Applications of Bioactive Compounds from Insects of the Family Blattidae Together with Related Drug Development. Molecules.

[47] Kucharzik T., Koletzko S., Kannengießer K., Dignaß A. (2020). Ulcerative Colitis—Diagnostic and Therapeutic Ralgorithms. Dtsch. Arztebl. Int..

[48] Thanikachalam P.V., Maurya R.K., Garg V., Monga V. (2019). An Insight into the Medicinal Perspective of Synthetic Analogs of Indole: A Review. Eur. J. Med. Chem..

[49] Cao R., Peng W., Wang Z., Xu A. (2007). β-Carboline Alkaloids: Biochemical and Pharmacological Functions. Curr. Med. Chem..

[50] Baek S.C., Nam K.H., Yi S.A., Jo M.S., Lee K.H., Lee Y.H., Lee J., Kim K.H. (2019). Anti-Adipogenic Effect of β-Carboline Alkaloids from Garlic (*Allium sativum*). Foods.

[51] Xing X., Li F., Hu Y., Zhang L., Hui Q., Qin H., Jiang Q., Jiang W., Fang C., Zhang L. (2022). Discovery of Novel Tetrahydro-β-Carboline Containing Aminopeptidase N Inhibitors as Cancer Chemosensitizers. Front. Oncol..

[52] Ma Z., Huang Y., Wan K., Zhu F., Sheng C., Chen S., Liu D., Dong G. (2021). Structural Simplification of Evodiamine: Discovery of Novel Tetrahydro-β-Carboline Derivatives as Potent Antitumor Agents. Bioorganic Med. Chem. Lett..

[53] Wang F.X., Deng A.J., Li M., Wei J.F., Qin H.L., Wang A.P. (2012). (3S)-1,2,3,4-Tetrahydro-β-Carboline-3-Carboxylic Acid from Cichorium Endivia. L Induces Apoptosis of Human Colorectal Cancer HCT-8 Cells. Molecules.

[54] Herraiz T. (1999). 1-Methyl-1,2,3,4-Tetrahydro-β-Carboline-3-Carboxylic Acid and 1,2,3,4-Tetrahydro-β-Carboline-3-Carboxylic Acid in Fruits. J. Agric. Food Chem..

[55] Herraiz T. (1998). Occurrence of 1,2,3,4-Tetrahydro-β-Carboline-3-Carboxylic Acid and 1-Methyl-1,2,3,4-Tetrahydro-β-Carboline-3-Carboxylic Acid in Fruit Juices, Purees, and Jams. J. Agric. Food Chem..

[56] Herraiz T., Galisteo J. (2003). Tetrahydro-β-Carboline Alkaloids Occur in Fruits and Fruit Juices. Activity as Antioxidants and Radical Scavengers. J. Agric. Food Chem..

[57] Herraiz T. (2000). Tetrahydro-Beta-Carboline-3-Carboxylic Acid Compounds in Fish and Meat: Possible Precursors of Co-Mutagenic Beta-Carbolines Norharman and Harman in Cooked Foods. Food Addit. Contam..

[58] Stavropoulou E., Pircalabioru G.G., Bezirtzoglou E. (2018). The Role of Cytochromes P450 in Infection. Front. Immunol..

[59] Kusunoki Y., Ikarashi N., Matsuda S., Matsukawa Y., Kitaoka S., Kon R., Tajima M., Wakui N., Ochiai W., Machida Y. (2015). Expression of Hepatic Cytochrome P450 in a Mouse Model of Ulcerative Colitis Changes with Pathological Conditions. J. Gastroenterol. Hepatol..

[60] Siewert E., Bort R., Kluge R., Heinrich P.C., Castell J.V., Jover R. (2000). Hepatic Cytochrome P450 Down-Regulation during Aseptic Inflammation in the Mouse Is Interleukin 6 Dependent. Hepatology.

[61] Whittaker C., Hynes R.O. (2002). Distribution and Evolution of von Willebrand/Integrin a Domains: Widely Dispersed Domains with Roles in Cell Adhesion and Elsewhere. Mol. Biol. Cell.

[62] Tadayon S.H., Vaziri-Pashkam M., Kahali P., Ansari Dezfouli M., Abbassian A. (2016). Common Genetic Variant in VIT Is Associated with Human Brain Asymmetry. Front. Hum. Neurosci..

[63] Nikolaus S., Schulte B., Al-Massad N., Thieme F., Schulte D.M., Bethge J., Rehman A., Tran F., Aden K., Häsler R. (2017). Increased Tryptophan Metabolism Is Associated with Activity of Inflammatory Bowel Diseases. Gastroenterology.

[64] Chen S., Wang M., Yin L., Ren W., Bin P., Xia Y., Liu G., Yang H., Tan B., Yin Y. (2018). Effects of Dietary Tryptophan Supplementation in the Acetic Acid-Induced Colitis Mouse Model. Food Funct..

[65] Derkacz A., Olczyk P., Olczyk K., Komosinska-Vassev K. (2021). The Role of Extracellular Matrix Components in Inflammatory Bowel Diseases. J. Clin. Med..

[66] Zhu J.J., Yao S., Guo X., Yue B.S., Ma X.Y., Li J. (2018). Bioactivity-Guided Screening of Wound-Healing Active Constituents from American Cockroach (*Periplaneta americana*). Molecules.

[67] Wang Y., Tang Q., Duan P., Yang L. (2018). Curcumin as a Therapeutic Agent for Blocking NF-ΚB Activation in Ulcerative Colitis. Immunopharmacol. Immunotoxicol..

[68] Lu P.D., Zhao Y.H. (2020). Targeting NF-ΚB Pathway for Treating Ulcerative Colitis: Comprehensive Regulatory Characteristics of Chinese Medicines. Chin. Med..

[69] Hegazy S.K. (2010). Effect of Probiotics on Pro-Inflammatory Cytokines and NF-ΚB Activation in Ulcerative Colitis. World J. Gastroenterol..

[70] Bantel H., Berg C., Vieth M., Stolte M., Kruis W., Schulze-Osthoff K. (2000). Mesalazine Inhibits Activation of Transcription Factor NF-ΚB in Inflamed Mucosa of Patients with Ulcerative Colitis. Am. J. Gastroenterol..

[71] Luo C., Zhang H. (2017). The Role of Proinflammatory Pathways in the Pathogenesis of Colitis-Associated Colorectal Cancer. Mediat. Inflamm..

[72] Stevens C., Walz G., Singaram C., Lipman M.L., Zanker B., Muggia A., Antonioli D., Peppercorn M.A., Strom T.B. (1992). Tumor Necrosis Factor-α, Interleukin-1β, and Interleukin-6 Expression in Inflammatory Bowel Disease. Dig. Dis. Sci..

[73] Ge C.Y., Wei L.Y., Tian Y., Wang H.H. (2020). A Seven-NF-ΚB-Related Gene Signature May Distinguish Patients with Ulcerative Colitis-Associated Colorectal Carcinoma. Pharmacogenomics Pers. Med..

[74] Shi L., Han X., Li J.X., Liao Y.T., Kou F.S., Wang Z.B., Shi R., Zhao X.J., Sun Z.M., Hao Y. (2020). Identification of Differentially Expressed Genes in Ulcerative Colitis and Verification in a Colitis Mouse Model by Bioinformatics Analyses. World J. Gastroenterol..

[75] Eiro N., Barreiro-Alonso E., Fraile M., González L.O., Altadill A., Vizoso F.J. (2022). Expression of MMP-2, MMP-7, MMP-9, and TIMP-1 by Inflamed Mucosa in the Initial Diagnosis of Ulcerative Colitis as a Response Marker for Conventional Medical Treatment. Pathobiology.

[76] Stallhofer J., Friedrich M., Konrad-Zerna A., Wetzke M., Lohse P., Glas J., Tillack-Schreiber C., Schnitzler F., Beigel F., Brand S. (2015). Lipocalin-2 Is a Disease Activity Marker in Inflammatory Bowel Disease Regulated by IL-17A, IL-22, and TNF-α and Modulated by IL23R Genotype Status. Inflamm. Bowel Dis..

[77] Bai X., Bai G., Tang L., Liu L., Li Y., Jiang W. (2020). Changes in MMP-2, MMP-9, Inflammation, Blood Coagulation and Intestinal Mucosal Permeability in Patients with Active Ulcerative Colitis. Exp. Ther. Med..

[78] Lin Q., Kang X., Li X., Wang T., Liu F., Jia J., Jin Z., Xue Y. (2019). NF-ΚB-Mediated Regulation of Rat CYP2E1 by Two Independent Signaling Pathways. PLoS ONE.

[79] Zhou M., Wang X., Shi H., Cheng L., Wang Z., Zhao H., Yang L., Sun J. (2016). Characterization of Long Non-Coding RNA-Associated CeRNA Network to Reveal Potential Prognostic LncRNA Biomarkers in Human Ovarian Cancer. Oncotarget.

[80] Chen Y., Tian Z., Hou H., Gai W. (2022). The Noncoding RNAs Regulating Pyroptosis in Colon Adenocarcinoma Were Derived from the Construction of a CeRNA Network and Used to Develop a Prognostic Model. BMC Medical Genom..

[81] Bian Y., Gao G., Zhang Q., Qian H., Yu L., Yao N., Qian J., Liu B., Qian X. (2019). KCNQ1OT1/MiR-217/ZEB1 Feedback Loop Facilitates Cell Migration and Epithelial-Mesenchymal Transition in Colorectal Cancer. Cancer Biol. Ther..

[82] Zhu M., Xie J. (2020). LncRNA MALAT1 Promotes Ulcerative Colitis by Upregulating LncRNA ANRIL. Dig. Dis. Sci..

[83] Zhang B., Li T., Wang C., Han J., Wang B., Sun G. (2020). LncRNA MALAT1: A Potential Therapeutic Target in DSSinduced Ulcerative Colitis Progression in Vitro. Trop. J. Pharm. Res..

[84] Wang Y., Wang N., Cui L., Li Y., Cao Z., Wu X., Wang Q., Zhang B., Ma C., Cheng Y. (2021). Long Non-Coding RNA MEG3 Alleviated Ulcerative Colitis through Upregulating MiR-98-5p-Sponged IL-10. Inflammation.

[85] Wang H., Teng J., Wang M., Zhang Y., Liu X., Liu Z. (2023). Expression and Significant Roles of the LncRNA NEAT1/MiR-493-5p/Rab27A Axis in Ulcerative Colitis. Immun. Inflamm. Dis..

