# Supplementary material for: Transcriptomic Insights into the Molecular Mechanisms of Indole Analogues from the Periplaneta americana Extract and Their Therapeutic Effects on Ulcerative Colitis
Source: Animals (Basel). 2024 Dec 30;15(1):63. doi: 10.3390/ani15010063 (PMC11718871; doi:10.3390/ani15010063)
Supplement: Supplementary file 1 [file animals-15-00063-s001.zip › animals-3343562-supplementary.pdf]

Supplementary Material

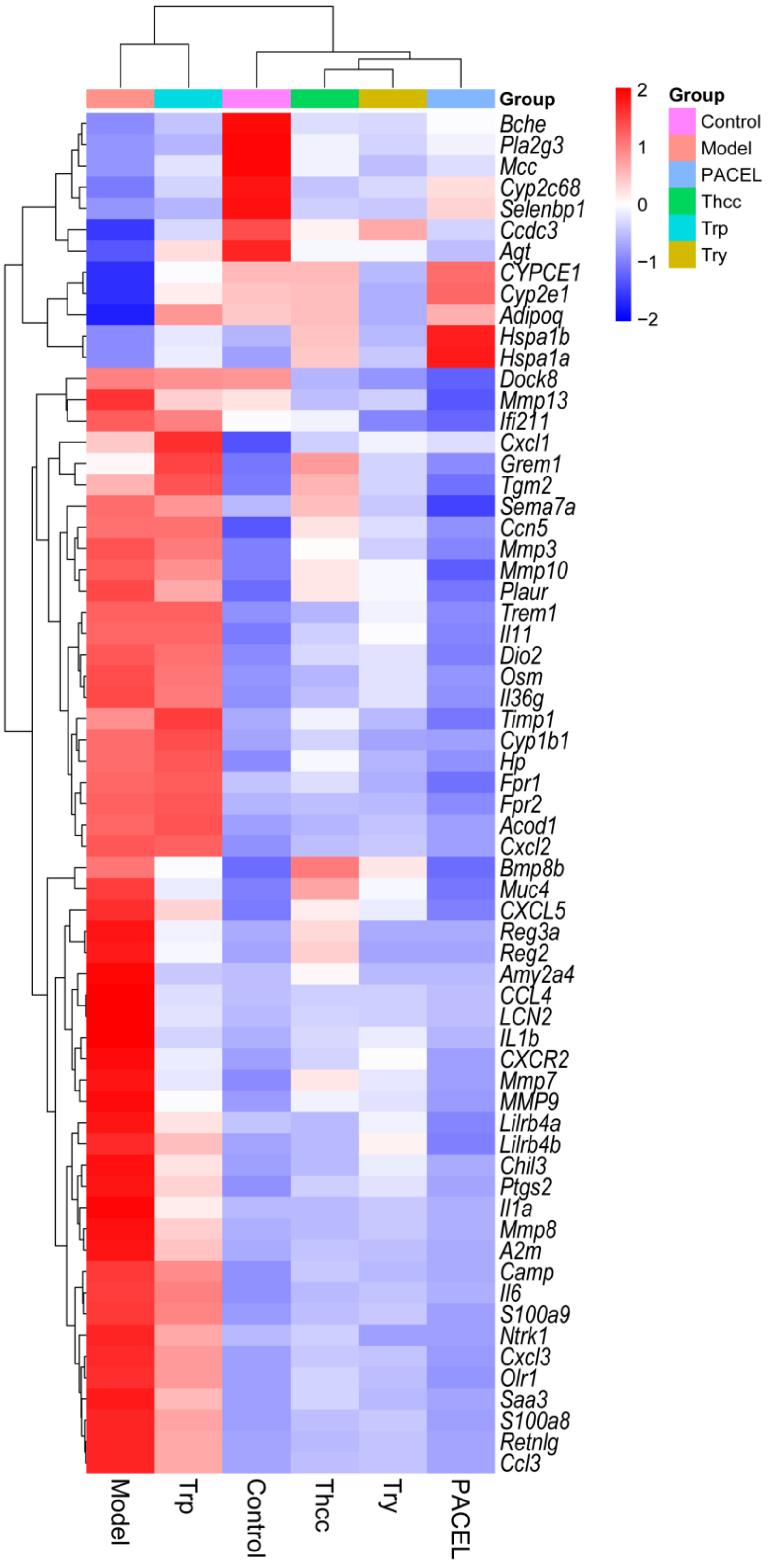

Figure S1. The heatmap of comparing DEGs expression profile.

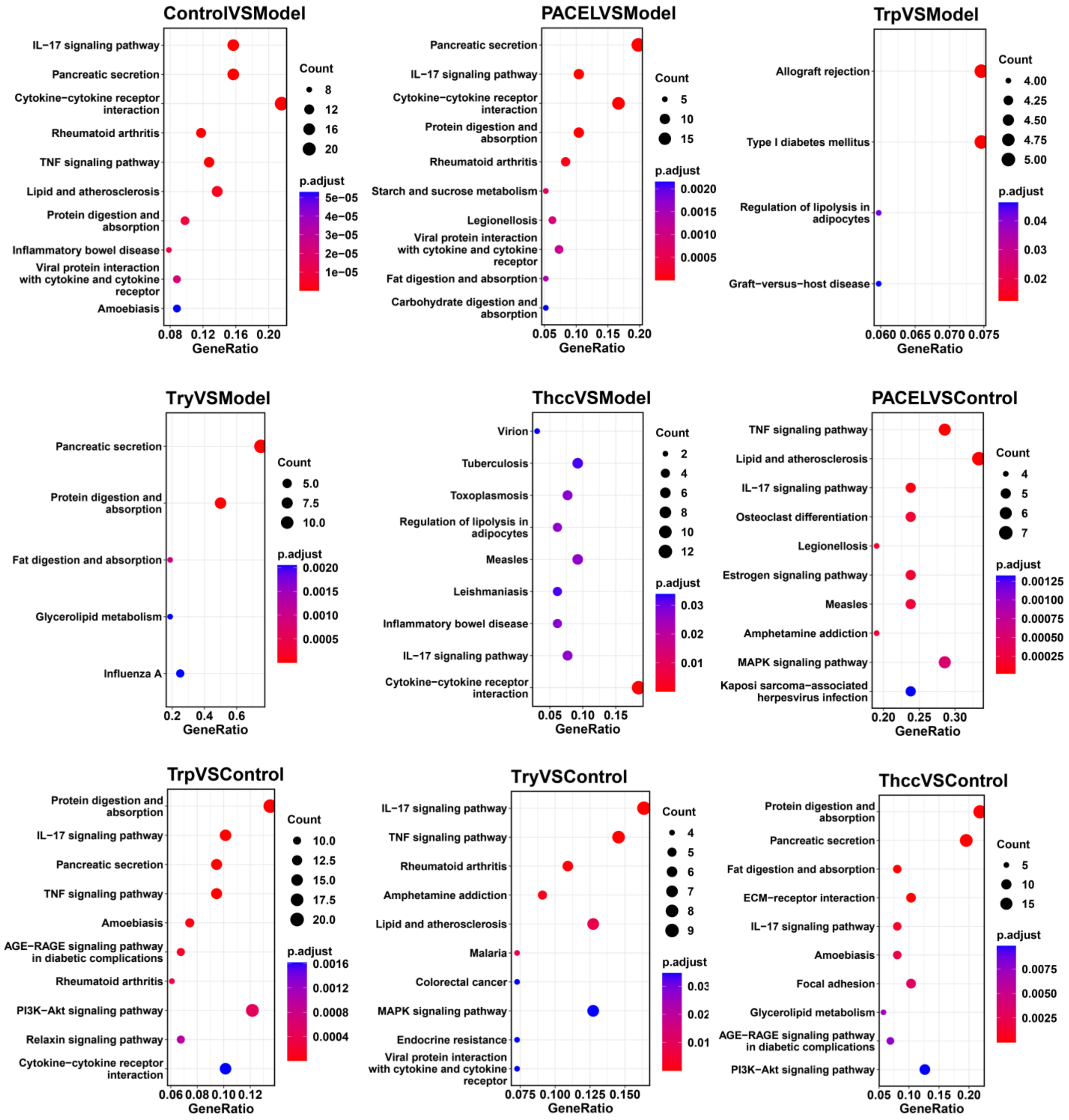

Figure S2. KEGG pathway enrichment of DEGs in different groups.

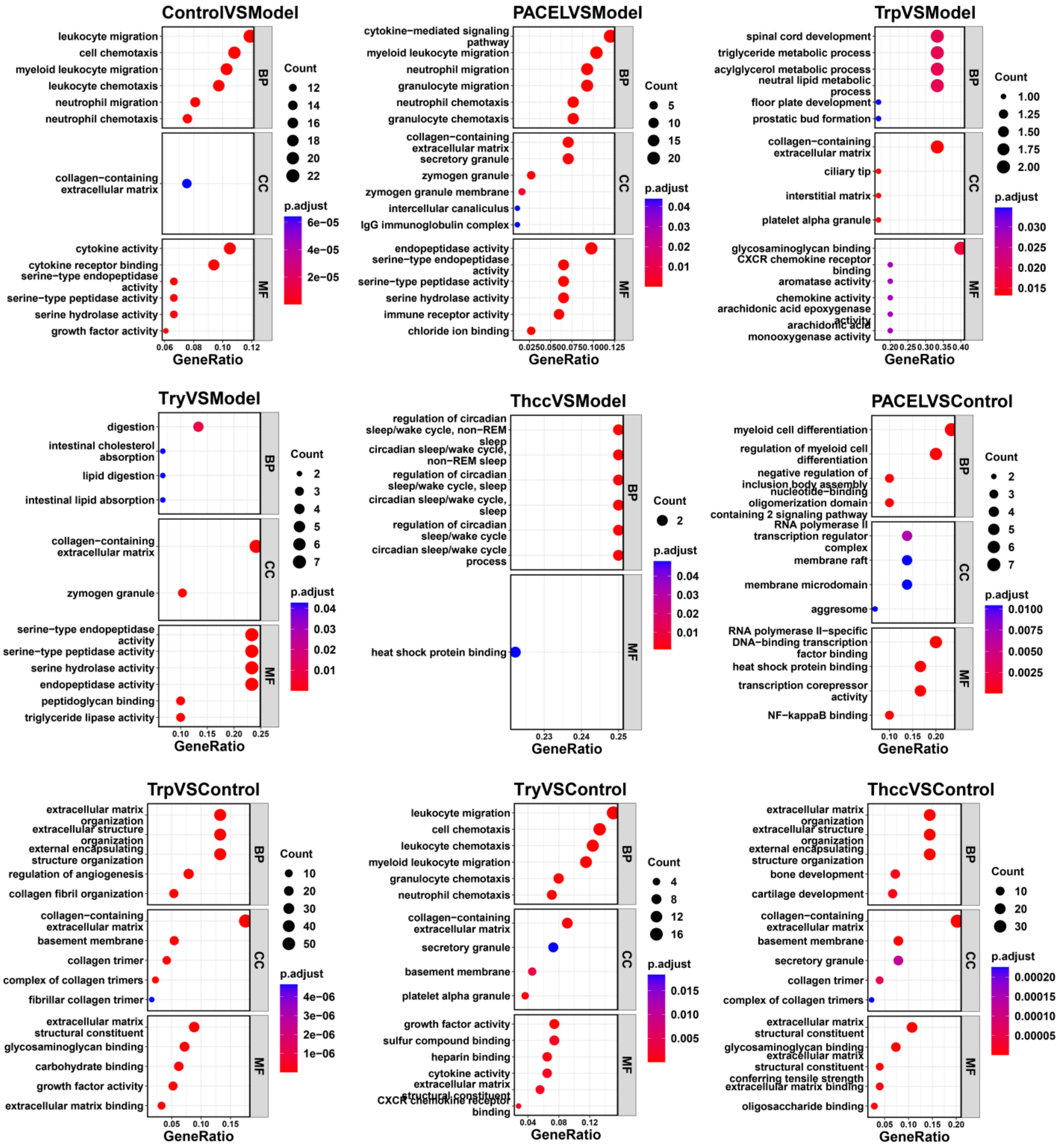

Figure S3. GO pathway enrichment of DEGs in different groups.

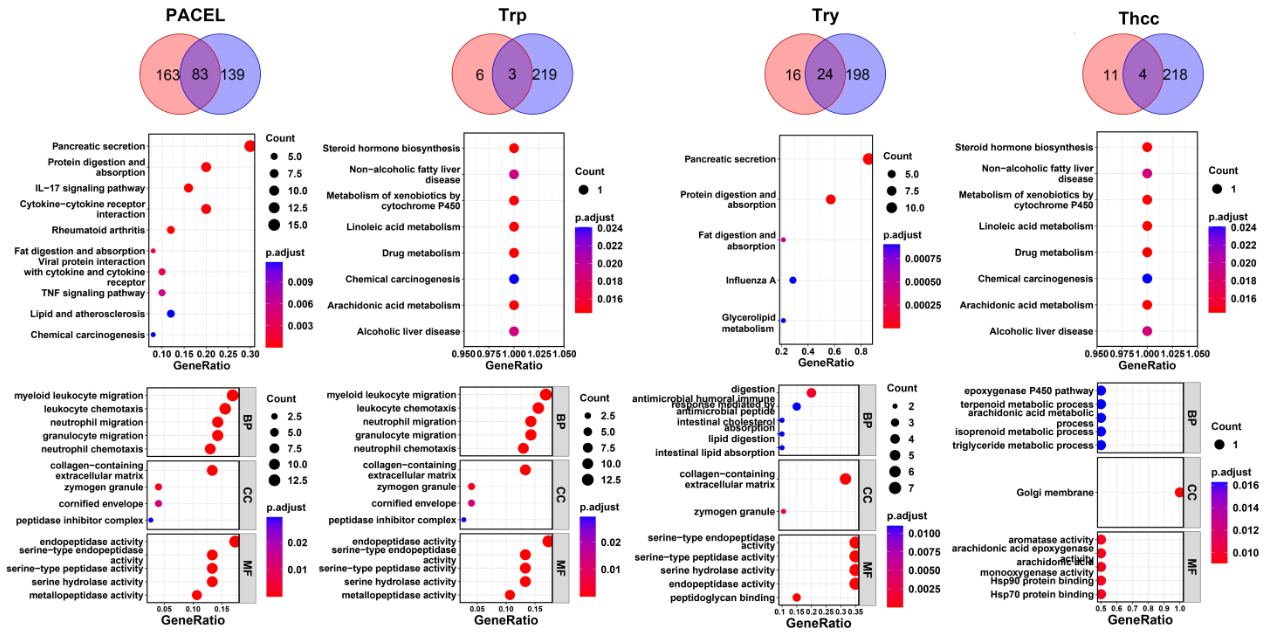

Figure S4. The KEGG (up) and GO (down) pathway enrichment of the shared DEGs in different treatment.

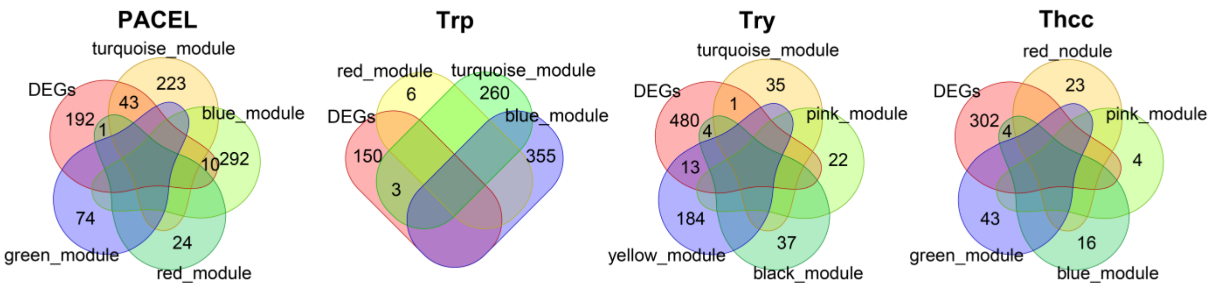

Figure S5. Venn diagram shows genes identified from the intersection of DEGs and significant module genes in WGCNA.

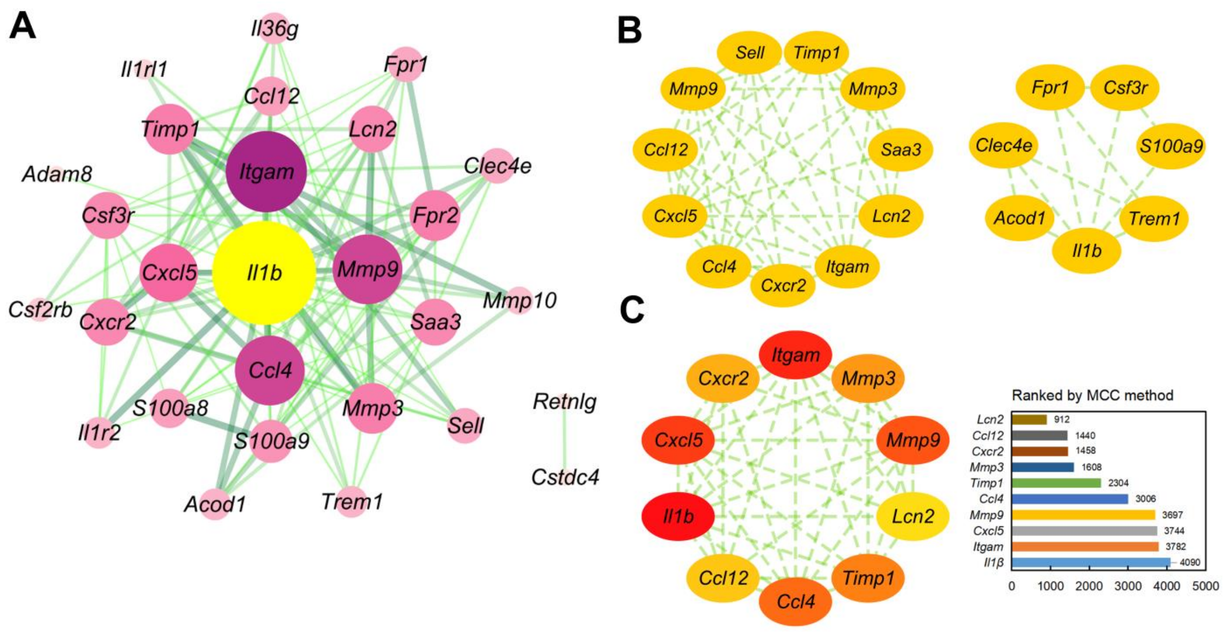

Figure S6. Protein interaction network. (A) PPI network. The nodes represent proteins. The edges represent their interaction. (B) MCODE sub-network, including cluster 1 and cluster 2. (C) Cytohubba-MCC was used to identify hub genes in the network.

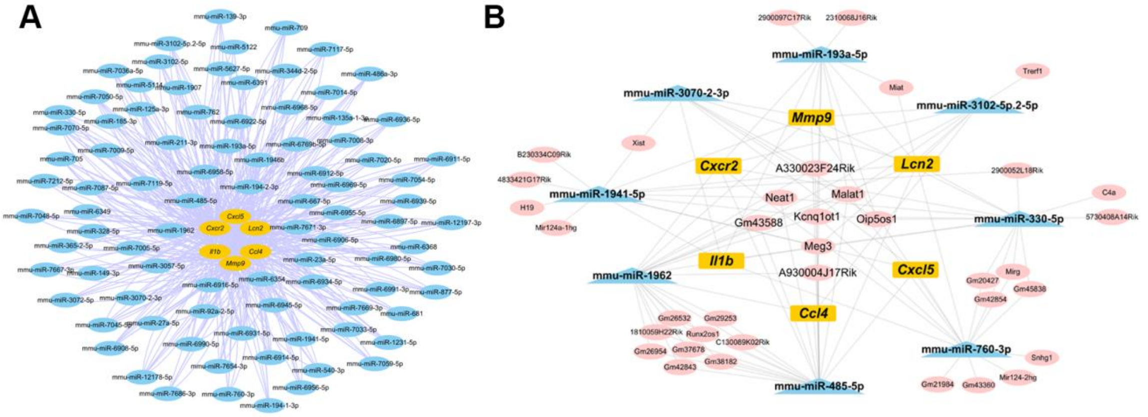

Figure S7. The integrated lncRNA-miRNA-hub genes network. (A) The miRNA-hubgenes network included 6 hub genes and 90 regulatory miRNAs. (B) The lncRNA-miRNA-hub genes network was constructed, including 8 lncRNA-miRNA subnetworks and the crossover points.

Table S1. Overlapping genes between DEGs and WGCNA module genes.

Group	Genes
PACEL	*9930111J21Rik2, Acod1, Adam8, Asprv1, Atg9b, Ccl12, Ccl4, Cd300lf, Clec4e, Cmah, Csf2rb, Csf3r, Cstdc4, Cxcl5, Cxcr2, ENSMUSG00000121093, F630028O10Rik, Fpr1, Fpr2, Gm19221, Gstm7, Hdc, Ighg2b, Ighg3, Igkj2, Igkj3, Igkj5, Igkv12-46, Il1b, Il1r2, Il1rl1, Il36g, Itgam, Lcn2, Lilrb4b, Mmp10, Mmp3, Mmp9, Nt5c1a, Otc, Rbp1, Retnlg, Runx1, S100a8, S100a9, Saa3, Selenbp1, Sell, Sema7a, Slc16a3, Stfa2l1, Timp1, Trem1, Tspan4*
Trp	*Fbn1, Eln, Mfap5*
Try	*Stx11, Slfn2, Ifit2, Rnf213, Rsad2, Thbs3, Mtss2, Foxf2, Twist1, Aspn, Cpxm2, Gucy1b1, Kcnd3, Plekhh2, Nkd2, Cped1, Tubb4a, S100b*
Thcc	*Gm15056, Gbp9, S1pr4, Sp110*
